# Leveraging electronic health records to study pleiotropic effects on bipolar disorder and medical comorbidities

**DOI:** 10.1038/tp.2016.138

**Published:** 2016-08-16

**Authors:** M L Prieto, E Ryu, G D Jenkins, A Batzler, M M Nassan, A B Cuellar-Barboza, J Pathak, S L McElroy, M A Frye, J M Biernacka

**Affiliations:** 1 Department of Psychiatry and Psychology, Mayo Clinic College of Medicine, Rochester, MN, USA; 2 Universidad de los Andes, Facultad de Medicina, Departamento de Psiquiatría, Santiago, Chile; 3 Department of Health Sciences Research, Mayo Clinic College of Medicine, Rochester, MN, USA; 4 Department of Psychiatry, Universidad Autónoma de Nuevo León, Nuevo León, Mexico; 5 Division of Health Informatics, Weill Cornell Medical College, Cornell University, New York, NY, USA; 6 Lindner Center of HOPE, Mason, OH, USA; 7 Department of Psychiatry and Behavioral Neuroscience, University of Cincinnati College of Medicine, Cincinnati, OH, USA

## Abstract

Patients with bipolar disorder (BD) have a high prevalence of comorbid medical illness. However, the mechanisms underlying these comorbidities with BD are not well known. Certain genetic variants may have pleiotropic effects, increasing the risk of BD and other medical illnesses simultaneously. In this study, we evaluated the association of BD-susceptibility genetic variants with various medical conditions that tend to co-exist with BD, using electronic health records (EHR) data linked to genome-wide single-nucleotide polymorphism (SNP) data. Data from 7316 Caucasian subjects were used to test the association of 19 EHR-derived phenotypes with 34 SNPs that were previously reported to be associated with BD. After Bonferroni multiple testing correction, *P*<7.7 × 10^−5^ was considered statistically significant. The top association findings suggested that the BD risk alleles at SNP rs4765913 in *CACNA1C* gene and rs7042161 in *SVEP1* may be associated with increased risk of ‘cardiac dysrhythmias' (odds ratio (OR)=1.1, *P*=3.4 × 10^−3^) and ‘essential hypertension' (OR=1.1, *P*=3.5 × 10^−3^), respectively. Although these associations are not statistically significant after multiple testing correction, both genes have been previously implicated with cardiovascular phenotypes. Moreover, we present additional evidence supporting these associations, particularly the association of the *SVEP1* SNP with hypertension. This study shows the potential for EHR-based analyses of large cohorts to discover pleiotropic effects contributing to complex psychiatric traits and commonly co-occurring medical conditions.

## Introduction

Patients with bipolar disorder (BD) have higher early mortality rates compared with the general population.^1, 2^ For example, a study of patients with BD from Scandinavian countries reported that their life expectancy was reduced by an average of 12.7 to 19.8 years among men and 11 to 16.2 years among women.^3^ Even though suicide is a well-known cause of death in this population, increasing evidence has shown a large contribution of medical illness, such as cardiovascular disease,^4, 5^ to premature mortality in patients with BD.^1^ The prevalence of medical comorbidities in this population is high,^6^ encompassing cardiovascular, neurovascular, neurologic and metabolic/endocrine diseases,^1, 7, 8, 9, 10^ and it is aggravated by the use of mood stabilizers and atypical antipsychotics, which are associated with weight gain, glucose disturbances and diabetes.^11, 12, 13^ Therefore, it is imperative to better understand the mechanisms underlying these phenomena to design interventions to treat and prevent these conditions among patients with BD.

BD is one of the most heritable psychiatric diseases, demonstrating 85% heritability in a large twin study.^14^ Genome-wide association studies (GWAS) have identified several genetic variants associated with BD risk,^15, 16, 17, 18^ including genes such as *CACNA1C*, *ODZ4*, *ITIH3–ITIH4*, *ANK3*, *SYNE1* and *TRANK1*. Moreover, many medical diseases frequently occurring in patients with BD are also complex heritable conditions.^19^ Thus, one potential mechanism underlying the increased prevalence of certain medical comorbidities in patients with BD may be pleiotropic effects of genetic variants associated with both BD and these medical conditions. For example, BD has been reported to be associated with the interaction between body mass index and variants in the *TCF7L2* gene, ^20^ which is a well-established type 2 diabetes risk gene.^21, 22^ Similarly, myocardial infarction risk has been reported to be associated with a genetic variant in the *ITIH3*–*ITIH4* genes,^23^ which has also been implicated in the risk for BD.^16^ Therefore, it is plausible that other common genetic variants may have pleiotropic effects, contributing to the risk for BD and its common medical comorbidities.^24^

Electronic health records (EHRs) are being increasingly utilized in research to identify patients with phenotypes of interest. When genetic data are available on large cohorts with EHR data, EHR-based phenotyping allows for testing for association of genetic variants with a wide range of medical conditions. The major advantage of this approach, which has been referred to as a ‘phenome-wide association study', or PheWAS, is that it provides a cost-effective approach to testing for association of genetic variants with multiple phenotypes.^25, 26, 27^ A recent study based on over 13 000 subjects reported that 66% of previously reported GWAS associations were replicated by this PheWAS approach, and several potential pleiotropic effects were identified.^25^ Following this EHR-based PheWAS approach, but focusing on previously reported BD risk single-nucleotide polymorphisms (SNPs) and selected key BD medical comorbidities (metabolic/endocrine, cardiovascular and neurological diseases), the current study aimed to test whether specific BD risk alleles are also associated with these medical illnesses.

## Materials and methods

### Study subjects

This study used the data from 7316 self-reported white subjects that are part of the Mayo Genomic Consortium (MayoGC).^28^ MayoGC is a cohort of patients from Mayo Clinic with longitudinal EHR data and existing genetic data from prior GWAS of various medical conditions, including peripheral arterial disease, venous thromboembolism, pancreatic cancer, brain cancer, lung cancer, ovarian cancer and chronic lymphocytic leukemia. For the cancer studies, disease cases are not included in the MayoGC.^28^ All subjects with data in the MayoGC gave general research consent to share genetic data with other researchers and to link the GWAS data with their EHR. This study was approved by the Mayo Clinic Institutional Review Board.

### Genotype data

The MayoGC participants were genotyped using various genotyping platforms as part of the original contributing studies, including the Illumina 660, 610, 550, and 370 (San Diego, CA, USA) and Affymetrix 6.0 (Santa Clara, CA, USA).^28^ For each study, standard quality control was performed. Briefly, SNPs with call rate <95% were removed, and subjects with missing genotype rates >5% were excluded. Subjects genotyped in more than one contributing study were identified and GWAS data with the highest call rate was retained for the analysis. All GWAS data was mapped to the forward strand and genome-wide imputation was performed using IMPUTE2 after pre-phasing the genotypes with the SHAPEIT program.^29^ The 1000 genomes cosmopolitan population was used as a reference panel for imputation, and imputed genotypes with imputation quality score (*r*^2^) of 0.3 or higher were included in the analysis.

### Phenotypes based on EHRs

All available EHR data from January 1994 to January 2015 were searched for all International Classification of Diseases Ninth Revision (ICD-9) codes. The ICD-9 codes were then classified into disease categories based on the Clinical Classifications Software (https://www.hcup-us.ahrq.gov/toolssoftware/ccs/ccs.jsp). Disease categories with strong epidemiological support of comorbidity with BD, excluding other mental disorders, (that is, metabolic/endocrine, cardiovascular and neurological diseases) were selected from all the CCS categories.^30, 31, 32, 33, 34, 35, 36, 37^ Nonspecific disorders (codes that include ‘other', such as ‘other circulatory disease') were excluded, owing to difficulty in interpreting association results for these phenotypes. Subjects with at least one ICD-9 code in the category were classified as cases, whereas remaining subjects were classified as controls. Finally, only phenotypes with prevalence of at least 5% in our sample were included in the analysis (Table 1).

### Selection of BD susceptibility SNPs

The National Human Genome Research Institute (NHGRI) catalog of published GWAS findings^38^ was used to identify 308 SNPs that were previously reported as associated with BD (accessed on July 2013). These SNPs were filtered using results from a mega-analysis of 7481 BD cases and 9250 controls performed by the Psychiatric Genomics Consortium (PGC),^15^ selecting only SNPs with *P*-values <10^−4^ in the mega-analysis. Of those SNPs supported by the PGC data, pairwise SNP linkage disequilibrium was calculated to identify SNP clusters in high linkage disequilibrium (*r*^2^⩾0.9). For each cluster, one SNP having the strongest association with BD risk in the PGC data was retained. This filtering strategy provided 34 SNPs with strong support for association with BD (Supplementary Table 1).

### Statistical analysis

Adjusting for contributing study and sex, logistic regression models were used to test the association between each SNP and each phenotype assuming log-additive allele effects. Odds ratios (ORs) for the effect of the minor allele on risk of the phenotype, and the corresponding nominal *P*-values are presented. After Bonferroni multiple testing correction, *P*<7.7 × 10^−5^ (=0.05/(34 × 19)) was considered statistically significant. All analyses were performed using R software (https://cran.r-project.org/).

### Replication

Although a direct replication cohort was not available, SNP-phenotype combinations corresponding to the top association signals were further examined using the PheWAS catalog (https://phewas.mc.vanderbilt.edu/), which represents a large collection of PheWAS results from the eMERGE network.^25, 39^ In addition, phenotypic and genetic data from the Mayo Clinic Bipolar Disorder Biobank^40, 41^ was used to examine the top SNP-phenotype combinations. Replication analyses using the Bipolar Biobank were limited to phenotypes available for BD Biobank subjects that were assessed using the Cumulative Illness Rating Scale (CIRS), which collects self-reported severity of various medical illness categories on an ordinal scale.^42^ The relevant phenotypes were dichotomized (none or no impairment vs at least mild problem), and logistic regression was used to test for association of the phenotype with the additively coded genotype, adjusting for sex.

## Results

The median age of the 7316 MayoGC subjects was 72 years old at the time of EHR-based phenotyping (or age at death), and 53% were male (Table 1). Among the 19 phenotypes included in the analysis, ‘disorders of lipid metabolism' was the most prevalent (75%), followed by ‘essential hypertension' (65%).

The top three SNP-phenotype association results are shown in Table 2. Among all combinations between the 19 phenotypes and 34 SNPs, the strongest association was observed between rs4765913 in the *CACNA1C* gene and the phenotype ‘cardiac dysrhythmias' (Table 2). The minor allele A of the SNP (minor allele frequency (MAF)=0.21), which was the risk allele for BD in the PGC analysis (OR=1.15, *P*=1.4 × 10^−6^), showed an increased risk effect for ‘cardiac dysrhythmias' (OR=1.13, *P*=3.4 × 10^−3^). Similar associations were observed between a protective BD allele (T) at SNP rs7042161 in the *SVEP1* gene and ‘essential hypertension' (OR=0.90; *P*=3.5 × 10^−3^). The SNP rs10896135 (MAF=0.26) in *C11orf80* gene also showed some evidence of association with risk of ‘headache including migraine' however, the protective BD allele of rs10896135 showed an increased risk effect for ‘headache including migraine', which suggests that this association is likely a false-positive. These associations are not significant after Bonferroni correction for multiple testing (threshold *P*=7.7 × 10^−5^).

The PheWAS catalog (https://phewas.mc.vanderbilt.edu/) was queried in an attempt to obtain additional evidence for the top observed SNP-phenotype associations. The *SVEP1* SNP with suggestive evidence of association with essential hypertension in our study is included in the online PheWAS catalog, with the results supporting our findings (*P*=0.0075 for association with hypertension; *P*=0.0086 for association with essential hypertension). The *CACNA1C* SNP with suggestive evidence of association with ‘cardiac dysrhythmias' in our study (rs4765913) was not included in the analyses performed by the eMERGE network that are documented in the PheWAS catalog. In fact, rs216013 is the only *CACNA1C* SNP in the PheWAS catalog results, and the catalog reports nominally significant evidence for association of this SNP with cardiac dysrhythmias (*P*=0.034). Because this SNP is in very low linkage disequilibrium with rs4765913 (*r*^2^=0.012), the potential association of rs216013 with cardiac dysrhythmias does not constitute replication of the result in the MayoGC sample, but it may provide additional evidence for a role of *CACNA1C* in this phenotype. The PheWAS catalog did not include any association results for SNPs in *C11orf80* gene; thus association of variation in this gene with headache/migraine could not be investigated using this resource.

Analysis of medical comorbidity phenotypes in the Mayo Clinic Bipolar Disorder Biobank precluded the replication of the associations between *CACNA1C* and cardiac dysrhythmias and between *C11orf80* and headache including migraine, as these phenotypes were not assessed in the biobank cohort. However, the CIRS was used to collect information on hypertension, with 644 of the genotyped BD subjects reporting none or no impairment related to hypertension, and 245 of the subjects reporting at least a mild problem related to hypertension. Comparison of these groups of patients provided independent marginal evidence for association of the *SVEP1* SNP rs7042161 with hypertension (OR=0.80, *P*=0.054 without sex adjustment; OR=0.82, *P*=0.082 after correction for sex).

## Discussion

This study applied a focused EHR-based ‘PheWAS' approach to explore genetic pleiotropic effects, focusing on genetic variants associated with BD risk and phenotypes that have been observed to co-occur with BD. The EHR-based ‘PheWAS' approach has been successfully applied in several large-scale studies, for example, by the Electronic Medical Records and Genomes (eMERGE) Network.^25^ A recent study demonstrated that an EHR-based approach produced nearly identical association signals to analysis of curated case–control data on hypothyroidism.^27^ Another study based on multiple populations in the National Health and Nutrition Examination Surveys identified 13 SNPs with evidence of pleiotropy.^43^ As more institutions are establishing biobanks for the purposes of biomedical research, and as the sample sizes of these collections increase, these types of studies will become more feasible and more powerful, providing the opportunity to investigate the genetic underpinnings of a variety of comorbid conditions. To our knowledge, this study represents the first application of the PheWAS approach, specifically in the context of investigating potential pleiotropic effects on BD risk and various comorbid medical conditions. We note that although recent efforts to study pleiotropy have often focused on determining total genome-wide genetic overlap between diseases,^44, 45, 46^ for example using polygenic risk score methods, the approach applied here is complementary to such efforts, as it attempts to identify specific SNPs that contribute to multiple diseases, in this case BD and comorbid medical conditions.

One finding of potential interest is the association between *CACNA1C* genetic variation and the ‘cardiac dysrhythmias' phenotype. SNPs in the *CACNA1C* gene are among the most replicated associations in GWAS of BD.^15^ Besides its role in the expression of calcium channels in the brain, the *CACNA1C* gene encodes voltage-gated calcium channels (CaV1.2) expressed in the cardiac myocites.^47^ Genetic variations in this gene have been reported to be associated with inherited arrhythmic disorders, involving shortened QTc interval, idiopathic ventricular fibrillation, early repolarization syndrome, J-wave syndromes, Brugada and Timothy syndromes and sudden cardiac death.^47, 48^ Mutations in *CACNA1C* gene have also been associated with long QTc interval syndrome, hypertrophic cardiomyopathy, congenital heart defects and sudden cardiac death.^49^ In Japanese patients mutations in this gene were associated with Brugada syndrome.^50^

It is therefore plausible that patients with BD that have alterations in the *CACNA1C* gene, may also express abnormal calcium channels in the heart, predisposing them to severe arrhythmias, particularly when exposed to mood stabilizers and antipsychotics which may impact the QTc interval; these calcium channel alterations may affect cardiac electrophysiology and may increase the risk for sudden cardiac death.^51^ If *CACNA1C* genetic variations are further confirmed to contribute to both BD and cardiovascular disease risk, it may impact treatment strategies, suggesting that perhaps calcium channel blockers should be investigated as mood stabilizers with cardiac protective effects for patients with BD.

The association between *SVEP1* (Sushi, Von Willebrand Factor Type A, EGF And Pentraxin Domain Containing 1) gene variants and essential hypertension also warrants attention. SVEP1 protein is composed of multiple domains: Sushi domain, von Willebrand factor type A domain, epidermal growth factor domain, hyaline repeat domain, and pentraxin domains.^52, 53^ The protein encoded by this gene has a role in several inflammatory mechanisms and cell adhesion processes; the sushi domain belongs to the family of complement control proteins and SVEP1 protein is similar to the family of selectin adhesion molecules. Although this gene is not believed to have a direct role in blood pressure control, it interacts with the *ICAM1* (intercellular adhesion molecule 1) gene, which has been implicated in several cardiovascular phenotypes, encompassing atherosclerosis, coronary artery disease and hypertension owing to its role in inflammatory processes.^54^ In particular, one study demonstrated an association between circulating levels of ICAM1 and blood pressure reduction in patients who underwent renal sympathetic denervation for refractory hypertension.^55^ Because the association between BD and hypertension has been well demonstrated,^10, 56^ this potential pleiotropic effect of a variant in *SVEP1* should be further investigated.

These results should be interpreted in light of the study's limitations. First, as in typical ‘PheWAS', phenotyping was solely based on EHR, and subjects with at least one ICD9 code in a given category were considered as cases, whereas remaining subjects made up the control group. Underreporting of cases can be expected in such EHR-based studies as the lack of a given ICD9 code in a health record does not necessarily indicate absence of the medical condition. Furthermore, because the EHR-derived codes include billing codes used for screening purposes, some false-positive case assignments are expected. However, we performed sensitivity analyses requiring at least two ICD9 codes to define a case, and the results were very similar (data not shown). Second, despite the fairly large overall sample, owing to the study design the number of cases for some of the phenotypes was relatively small (for example, 520 cases of ‘acute myocardial infarction'), providing limited power for some of the analyses. Third, because the study did not include patients with BD, we were not able to test for pleiotropic effects in patients with both BD and the medical diseases we studied. Finally, the lack of a direct replication sample is a limitation, and further replication will be important to confirm the observed associations. Nevertheless, the additional results from the PheWAS catalog and the Mayo Clinic Bipolar Disorder Biobank lend some support for the top two observed associations.

In conclusion, this study illustrates the application of an EHR-based genetic ‘PheWAS' approach to the investigation of the pleiotropic effects contributing to medical comorbidities of psychiatric illness. With the increasing availability of genetic data, such studies are beginning to have a prominent role in biomedical research, and may make a significant contribution to the understanding of complex genetic diseases.

## Figures and Tables

**Table 1:** Demographic characteristics of study subjects and frequencies of 19 phenotypes

*Demographic characteristics and phenotypes*	*Study subjects (*n=*7316)*
Age, median (25th–75th percentiles)	72 (63–80)
Sex, male *N* (%)	3843 (53%)
	
*Phenotypes,* N *(%)*	
Metabolic/endocrine	
Disorders of lipid metabolism	5468 (75%)
Diabetes mellitus	3941 (46%)
Thyroid disorders	1916 (26%)
	
* *Cardiovascular	
Essential hypertension	2564 (65%)
Cardiac dysrhythmias	3509 (52%)
Coronary atherosclerosis and other heart disease	2947 (40%)
Peripheral visceral atherosclerosis	2907 (40%)
Heart valve disorders	2519 (34%)
Conduction disorders	1937 (26%)
Occlusion or stenosis of precerebral arteries	1144 (16%)
Aortic, peripheral and visceral artery aneurysms	1078 (15%)
Congestive heart failure; nonhypertensive	848 (12%)
Acute cerebrovascular disease	649 (8.9%)
Peri-, endo- and myocarditis, cardiomyopathy	614 (8.4%)
Aortic and peripheral arterial embolism or thrombosis	603 (8.2%)
Acute myocardial infarction	520 (7.1%)
Transient cerebral ischemia	485 (6.6%)
	
* *Neurological	
Headache including migraine	1810 (25%)
Epilepsy; convulsions	711 (9.7%)

**Table 2:** Top three most significant associations between BD-associated SNPs and EHR-derived phenotypes

*SNP*	*Gene*	*chr*	*Position*	*Minor allele*	*MAF*	*OR for BD risk*^a^	P*-value for BD risk*^a^	*Phenotype*	*OR*^b^	P*-value*^b^
rs4765913	*CACNA1C*	12	2290157	A	0.21	1.15	1.4 × 10^−6^	Cardiac dysrhythmias	1.13	3.4 × 10^−3^
rs7042161	*SVEP1*	9	112237084	T	0.34	0.90	7.3 × 10^−5^	Essential hypertension	0.90	3.5 × 10^−3^
rs10896135	*C11orf80*	11	66307578	C	0.26	0.88	1.8 × 10^−6^	Headache including migraine	1.12	8.5 × 10^−3^

Abbreviations: BD, bipolar disorder; EHR, electronic health record; MAF, minor allele frequency; OR, odds ratio; SNP, single-nucleotide polymorphism.

a OR and *P*-value for the minor allele from the Psychiatric Genomics Consortium BD GWAS.^15^

b OR and *P*-value for the association between the SNP minor allele and the phenotype.
