# Supplementary material for: Leveraging electronic health records to study pleiotropic effects on bipolar disorder and medical comorbidities
Source: Transl Psychiatry. 2016 Aug 16;6(8):e870–. doi: 10.1038/tp.2016.138 (PMC5022084; doi:10.1038/tp.2016.138)
Supplement: Supplementary Table 1 [file tp2016138x1.doc]

**Supplementary Table 1**: 34 Bipolar disorder - associated SNPs analyzed in the study. Odds ratio (OR; for allele 1) and p-values are for association between each SNP and BD risk from the PGC BD GWAS ^1^ available through [www.med.unc.edu/pgc/downloads](http://www.med.unc.edu/pgc/downloads).

| Chr | SNP | Gene | Position | Allele1 | Allele2 | Allele 1 Freq^*^ | | OR^**^ | | P-values | |
| --- | --- | --- | --- | --- | --- | --- | --- | --- | --- | --- | --- |
| 1 | rs4660531 | FOXO6 | 41612409 | G | T | 0.69 | 0.89 | | 8.24E-06 | |  |
| 1 | rs4650608 | LOC652549 | 79010603 | C | T | 0.32 | 0.90 | | 2.00E-05 | |  |
| 1 | rs12730292 | LOC652549 | 79027350 | C | G | 0.66 | 1.12 | | 5.93E-06 | |  |
| 2 | rs3845817 | RPS15AP15 | 65612029 | C | T | 0.60 | 0.90 | | 3.92E-06 | |  |
| 2 | rs6746896 | LMAN2L | 96774676 | A | G | 0.67 | 1.14 | | 4.20E-07 | |  |
| 2 | rs7578035 | C2orf55 | 98749324 | G | T | 0.48 | 1.12 | | 4.42E-06 | |  |
| 2 | rs6733011 | C2orf55 | 98831934 | A | G | 0.47 | 0.91 | | 7.28E-05 | |  |
| 2 | rs2176528 | GLULP6 | 1.95E+08 | C | G | 0.75 | 1.15 | | 1.07E-05 | |  |
| 3 | rs9834970 | TRANK1 | 36831034 | C | T | 0.51 | 1.11 | | 6.19E-06 | |  |
| 3 | rs6550435 | TRANK1 | 36839493 | G | T | 0.35 | 1.12 | | 4.80E-06 | |  |
| 3 | rs10865974 | PBRM1 | 52693320 | G | T | 0.56 | 1.12 | | 1.22E-06 | |  |
| 3 | rs1042779 | ITIH1 | 52796051 | A | G | 0.60 | 1.13 | | 1.90E-06 | |  |
| 3 | rs2535629 | ITIH3 | 52808259 | A | G | 0.38 | 0.88 | | 8.20E-07 | |  |
| 3 | rs736408 | ITIH3 | 52810394 | C | T | 0.61 | 1.14 | | 2.00E-07 | |  |
| 4 | rs360932 | LOC100505685 | 1.53E+08 | A | G | 0.66 | 1.10 | | 5.11E-05 | |  |
| 6 | rs9371601 | SYNE1 | 1.53E+08 | G | T | 0.64 | 0.87 | | 4.33E-09 | |  |
| 6 | rs7747960 | SYNE1 | 1.53E+08 | A | C | 0.15 | 1.15 | | 7.69E-05 | |  |
| 7 | rs4332037 | MAD1L1 | 1917335 | C | T | 0.81 | 0.87 | | 4.28E-06 | |  |
| 7 | rs7799006 | FTSJ2 | 2244752 | C | T | 0.66 | 1.11 | | 4.49E-05 | |  |
| 7 | rs2398668 | SNX8 | 2264753 | C | T | 0.66 | 1.13 | | 1.16E-05 | |  |
| 8 | rs7827290 | LOC731779 | 1.42E+08 | G | T | 0.37 | 1.13 | | 9.36E-06 | |  |
| 9 | rs7042161 | SVEP1 | 1.12E+08 | C | T | 0.67 | 1.11 | | 7.31E-05 | |  |
| 9 | rs2905072 | GFI1B | 1.35E+08 | A | G | 0.19 | 1.15 | | 5.01E-05 | |  |
| 10 | rs10994397 | ANK3 | 61949130 | C | T | 0.91 | 0.74 | | 5.54E-10 | |  |
| 11 | rs10896135 | C11orf80 | 66307578 | C | G | 0.25 | 0.88 | | 1.83E-06 | |  |
| 11 | rs12576775 | ODZ4 | 78754841 | A | G | 0.82 | 0.85 | | 2.66E-08 | |  |
| 11 | rs12290811 | ODZ4 | 78761268 | A | T | 0.14 | 1.19 | | 9.25E-08 | |  |
| 12 | rs1006737 | CACNA1C | 2215556 | A | G | 0.32 | 1.11 | | 1.73E-05 | |  |
| 12 | rs4765913 | CACNA1C | 2290157 | A | T | 0.24 | 1.15 | | 1.35E-06 | |  |
| 12 | rs2070615 | CACNB3 | 47504438 | A | G | 0.40 | 0.90 | | 1.08E-05 | |  |
| 12 | rs10860392 | ANKS1B | 98022318 | C | T | 0.39 | 0.91 | | 9.32E-05 | |  |
| 14 | rs12436436 | C14orf165 | 23451355 | C | T | 0.09 | 1.20 | | 2.87E-05 | |  |
| 15 | rs12912251 | C15orf53 | 36773660 | G | T | 0.74 | 1.13 | | 2.10E-06 | |  |
| 19 | rs2287921 | RASIP1 | 53920084 | C | T | 0.47 | 1.12 | | 4.00E-06 | |  |

^*^ Frequency of allele 1 obtained from the 1000 genome EUR population

^**^ OR for Allele 1

References:

^1^ Psychiatry GWAS consortium bipolar disorder working group. Large genome-wide association analysis of biplar disorder identifies a new susceptibility locus near ODZ4. Nature Genetics 2011. 43, 977-983.
